# Forty Years Ago

**Published:** 1884-07

**Authors:** 


### FORTY YEARS AGO.

Coffins were very plain and burial caskets were unknown.

Tombstones had larger epitaphs and more verbosity engraved upon them.

Eggs were a shilling a dozen and butter was considered high at eighteen cents per pound.

Much of the silver currency, fips, levies and dollars was of Mexican and Spanish coinage.

The country retail trade was much better, as people could not so easily run to the city by rail.

Business letters were more voluminous and formal, and were written in a precise, round hand.

There was York currency, eight shillings to the dollar, and New England currency, six shillings to the dollar.

The diet was more surcharged with grease, the winter breakfast usually being made of salted ham and hot cakes.

New Orleans and Muscovado molasses, very black and thin, was the common sweetening for buckwheat cakes. Refined molasses was almost unknown.

The bank bills were of State banks, and the further West their locality the shakier they were. Illinois and Indiana bills would barely pass in New York.

Bread was home made. Coffee was freshly ground every morning, and the grinding of the family coffee mill was a familiar sound before the children arose.

Negro minstrelsy was just cropping out in the travelling circus. There were generally but two performers, who assumed male and female characters. The popular melody was "Jump Jim Crow."

